# Intravascular injection of colored molding materials for human anatomy teaching: a literature review

**DOI:** 10.1007/s00276-026-03938-3

**Published:** 2026-07-14

**Authors:** Sami Schranz, Hugues Cadas, Sara Sabatasso

**Affiliations:** Faculty Unit of Anatomy and Morphology (UFAM), University Center of Legal Medicine Lausanne-Geneva (CURML), Lausanne University Hospital (CHUV), University of Lausanne, Lausanne, Switzerland

**Keywords:** Anatomy teaching, Anatomical dissection, Intravascular injection, Molding material, Post mortem angiography, Educational technology

## Abstract

**Purpose:**

Intravascular injection of colored molding materials used either alone or in combination with radiological contrast agents is increasingly used to enhance vascular visualization in cadaveric specimens for anatomical education and surgical training. Published studies remain scattered and show substantial methodological variation. This structured literature review summarizes current practices regarding specimen preparation, injection techniques, casting materials, contrast agents, and imaging modalities, with particular emphasis on their educational applications.

**Materials and methods:**

A comprehensive literature search was conducted in PubMed, Scopus, Embase, Web of Science, and Google Scholar (1975–August 2025) using English, French, and German keywords. Reference lists were screened manually. After removal of duplicates, 702 records were screened, 145 full-text articles assessed, and 78 studies included.

**Results:**

Included studies demonstrated variability in casting materials (primarily latex, with silicone, epoxy, and acrylic resins also reported), contrast agents (iodinated and barium-based compounds and lead oxide for CT, with gadolinium-based formulations predominantly used for MRI), perfusion pressures, flushing strategies, and specimen conditions (fresh-frozen, formalin-fixed, and Thiel-embalmed). However, methodological heterogeneity and inconsistent reporting limited direct comparison between approaches. The review nevertheless identifies representative reported technical protocols and highlights the main practical variables influencing protocol selection.

**Conclusion:**

Contrast-enhanced vascular casting provides valuable tools for anatomy education and surgical training by enabling direct correlation between radiological datasets and anatomical dissection. Beyond summarizing the literature, this review also offers practical protocol-selection guidance according to educational, research, and imaging objectives. As such, it may serve as a useful reference for teams implementing or refining these techniques in modern teaching environments.

**Supplementary Information:**

The online version contains supplementary material available at 10.1007/s00276-026-03938-3.

## Introduction

A detailed understanding of vascular anatomy is essential to both medical education and clinical practice. Traditional cadaveric dissection has long been the foundation of anatomical training, offering hands-on experience and a physical connection to the human body. This method presents limitations, especially in the study of the vascular system. Blood vessels often collapse post-mortem, and their lack of color differentiation complicates the identification of arterial and venous pathways, particularly in smaller or deeply situated structures [18].

To overcome these limitations, intravascular injection techniques have been developed, using colored molding materials such as latex, silicone, and resin. These materials are injected into the vascular system of cadavers, where they harden and create a cast of the vascular architecture [11, 36]. Red and blue dyes are typically used to distinguish arteries from veins, enhancing visibility during dissection. The resulting models preserve the three-dimensional layout of the vascular network and allow for repeated use in teaching settings. This technique also facilitates a more intuitive understanding of complex vascular routes. Nevertheless, it requires technical expertise and careful execution to ensure uniform perfusion and avoid artifacts or vessel damage.

A significant evolution in this field has been the integration of radiological contrast agents with molding compounds [18, 27, 30], enabling the use of imaging modalities such as X-ray, computed tomography (CT), and magnetic resonance imaging (MRI). This approach, as post-mortem angiography, offers high-resolution, three-dimensional visualization of the vasculature and provides valuable correlations with clinical imaging. When combined with dissection, these techniques enrich the anatomical learning experience and help bridge the gap between radiological findings and gross anatomy. Unlike in vivo imaging approaches such as CTA or MRA, cadaveric vascular injection models offer a unique advantage for anatomical education and surgical training, as the same specimen can be injected, imaged, and subsequently dissected. This allows direct correlation between radiological datasets and anatomical structures during dissection, enabling detailed exploration of three-dimensional vascular relationships. Their application has also expanded to the development of virtual models, useful in surgical simulation and digital education platforms.

Historically, the injection of substances into anatomical specimens has a long tradition. In the seventeenth century, anatomists experimented with wax [37] or dye [13] injections to better visualize internal structures. Though pioneering for their time, these techniques were limited in precision and durability. The mid-twentieth century saw the rise of latex-based compounds, which were more flexible and easier to handle. In recent decades, silicone and epoxy resins have become popular due to their stability, and compatibility with advanced imaging contrast media and technologies. These innovations have broadened the scope of anatomical modeling and improved the fidelity of vascular reproductions [8, 11, 25, 31].

Despite their advantages, such techniques have limitations. Technical challenges include ensuring complete and even perfusion, avoiding vascular ruptures, and managing potential imaging artifacts. Furthermore, the costs and limited access to imaging equipment may restrict its use in some institutions, limiting the widespread adoption of these methods.

In recent years, interest in these techniques has grown considerably due to their educational benefits. They enhance student engagement and spatial understanding, especially when radiological and anatomical data are integrated. The potential for combining these models with virtual reality (VR) or augmented reality (AR) also represents a promising avenue for remote and interactive learning.

This literature review aims to provide a comprehensive analysis of intravascular injection techniques using colored molding materials with or without radiological contrast agents for anatomical education and research. It explores their historical development, methodological diversity, imaging modalities, and anatomical applications. Focus is placed on specimen preparation, material compatibility with imaging, and integration into educational practices. Moreover, this review aims to support educators and researchers in selecting effective injection and imaging protocols, while highlighting current limitations and future directions to enhance anatomical visualization, surgical simulation, and multimodal teaching strategies. Because methodological information remains fragmented across anatomical, surgical, and radiological publications, this review also seeks to organize the available technical knowledge and highlight current practices and methodological variability reported in the literature. By synthesizing these data, the review aims to provide both a structured reference framework and a more practical orientation for teams implementing vascular injection models combined with modern imaging technologies in anatomy teaching and surgical training. To this end, representative reported protocols are summarized in a dedicated technical table, and a second table proposes protocol-selection options according to intended educational, research, and imaging objectives.

## Material and methods

### Review methodology

This literature review was designed according to the Preferred Reporting Items for Systematic Reviews and Meta-Analyses (PRISMA) guidelines. The objective was to identify and synthesize studies describing intravascular injection of colored molding materials in human cadaveric specimens, whether used alone or combined with radiological contrast agents, in the context of anatomical education, surgical training, and anatomical research.

### Search strategy

Five electronic databases, PubMed, Scopus, Embase, Web of Science and Google Scholar, were searched using the same combinations of the following keywords: “intravascular injection,” “colored molding,” “radiological contrast media,” “anatomy,” and “anatomical education.” Equivalent French (“injection intravasculaire,” “moulage vasculaire,” “produit de contraste”) and German terms (“intravasale Injektion,” “Gefäßausguss,” “Kontrastmittel”) were added to broaden retrieval of non-English publications. Boolean operators (AND, OR) were used. The search covered the period from 1975 to August 2025, and the last electronic search was conducted on August 15, 2025. Reference lists of included articles were also screened.

### Inclusion and exclusion criteria

Inclusion criteria were: 1. studies using intravascular injection of colored molding materials (e.g., latex, silicone, resin) and/or radiological contrast agents; 2. use of imaging modalities (CT, MRI, X-ray, or hybrid) or post-injection non-radiological documentation methods such as gross photography and microscopy, to assess injected vascular structures in human cadavers; 3. reporting of educational, anatomical, or surgical outcomes; 4. reporting of casting materials, contrast agents, imaging methods, or technical workflow characteristics; 5. publication in English, French, or German in peer-reviewed journals. Exclusion criteria were: 1. letters, editorials, or conference abstracts; 2. duplicate publications; 3. in vivo imaging studies without postmortem applications; 4. studies conducted on animal models or other non-human specimens; 5. studies not reporting relevant educational, surgical or anatomical outcomes.

Studies without radiological imaging were retained only when post-injection photographic or microscopic documentation provided technical information directly relevant to contrast-enhanced vascular injection workflows in human cadaveric specimens.

### Study selection and data extraction

Titles and abstracts were screened, and full texts of eligible articles were reviewed by a single author. Potential uncertainties were addressed by rechecking inclusion criteria and cross-verifying extracted data to ensure consistency.

 Data were extracted for: 1. authors and year; 2. specimen type and number; 3. imaging modality; 4. casting material; 5. preservation method; 6. vascular flushing technique; 7. anatomical focus; 8. use of 3D imaging or printing; 9. integration in teaching; and 10. first author’s specialty.

### Data synthesis

A descriptive synthesis was performed, analyzing publication trends, imaging modality usage, materials, and educational application. Author specialties were analyzed to assess interdisciplinary involvement. Percentages presented in the results and tables were calculated based on the total number of included studies (*n* = 78).

### PRISMA flow diagram

**Fig. 1:**
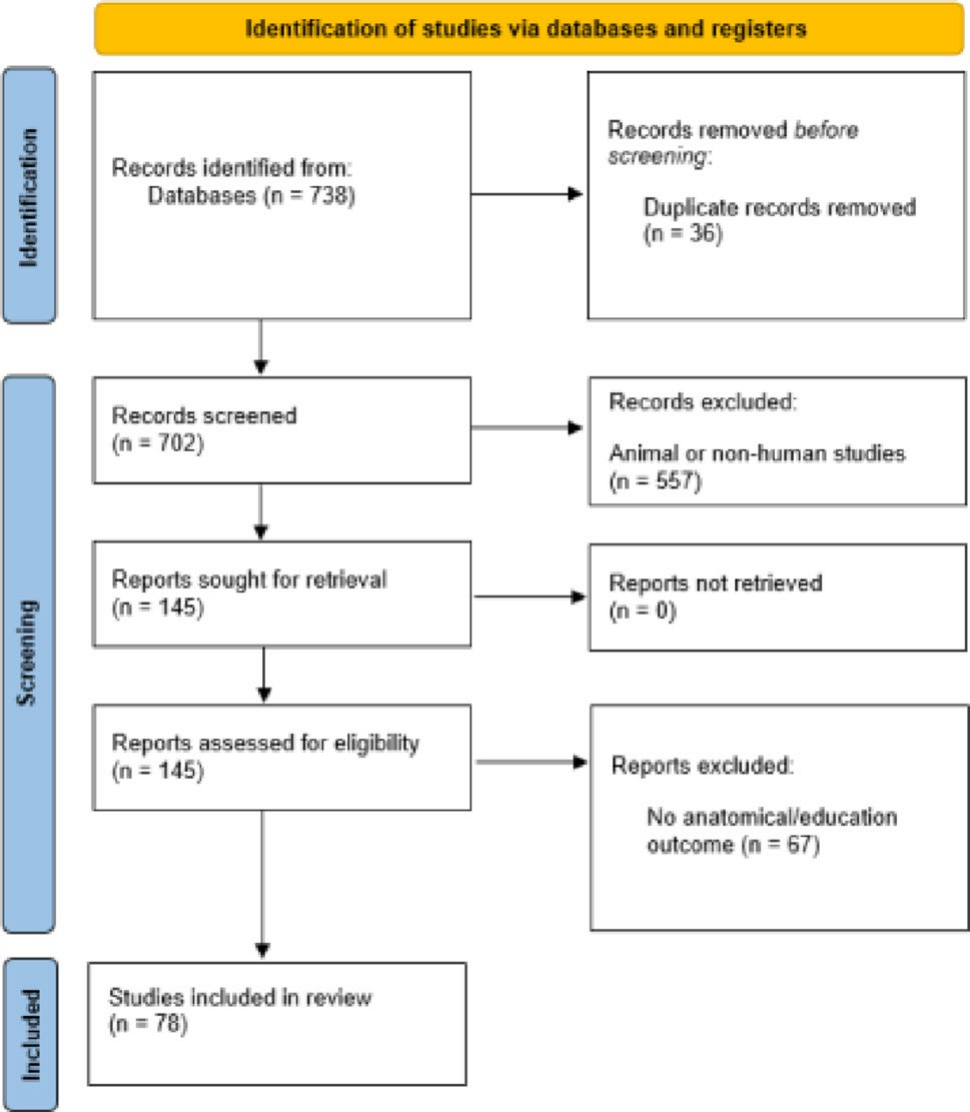
PRISMA 2020 flow diagram illustrating the study selection process

A total of 738 articles were initially retrieved. After duplicate removal and screening, 78 studies met the inclusion criteria. The full selection process is illustrated in Fig. 1 using a PRISMA 2020 flow diagram.

A complete list of all studies included in the review is provided in Online Resource 1.

## Results

### Publication trends and research evolution

The analysis of the 78 reviewed articles revealed an increase in publications over the past two decades (Table 1). This surge coincides with advancements in imaging technologies and their increasing integration into clinical practice. Early studies, such as that of Plaisant et al. [27], emphasized the technical feasibility of combining contrast media injections with CT and MRI imaging. Although these studies demonstrated the potential of imaging to enhance anatomical understanding, they were constrained by the limitations of available imaging modalities. At the time, CT imaging lacked submillimetric resolution, with voxel sizes that limited the visualization of fine vascular structures. Similarly, MRI acquisitions were performed on 1.5 T systems, which offered lower signal-to-noise ratios and reduced vascular contrast compared to modern 3 T or 7 T MRI [22, 28].

**Table 1:** Article occurrence over time across the reviewed studies

Year range	Number of articles
1975–1989	5
1990–1999	12
2000–2009	20
2010–2019	25
2020–2025	16

In comparison, more recent research [4], illustrates how technological advancements have enabled the creation of high-resolution, three-dimensional vascular models using advanced imaging techniques, including 3 T MRI and dedicated 3D sequences. This reflects a natural progression where improved imaging capabilities build upon prior methodological refinements.

### Specimen preservation methods

The studies reviewed underscore the influence of specimen preservation methods on the quality of vascular injections and subsequent imaging (Table 2). Formalin-fixed specimens exhibit reduced vascular permeability due to fixation-induced tissue cross-linking [11], a phenomenon absent in fresh or frozen cadavers, which facilitates more homogeneous contrast agent distribution during vascular injection.

**Table 2:** Type of tissue used across the reviewed studies

Preservation method	Number of articles	Percentage of total (%)	Injection before fixation	Means of preservation
Fresh	20	25.64%	Not applicable	Not applicable
Frozen	25	32.05%	Not applicable	Freezing
Fixed (Embalmed)	33	42.31%	18 (54.55%)	Formaldehyde or Thiel solution

Yet, specimens embalmed using Thiel’s method, combined with latex-based vascular injection, have demonstrated preserved soft tissue pliability and excellent contrast visualization in CT imaging [35]. In a study utilizing Thiel-embalmed cadaveric heads with latex milk injection under controlled perfusion (0.5 bar) a detailed radiographic visualization of vascular structures, including intraosseous and periosteal anastomoses is shown (Fig. 4). Thiel-embalmed specimens with latex injection offer an optimal balance between soft tissue preservation and CT vascular imaging.

### Vascular flushing techniques

The studies reviewed show the importance of vascular flushing as a preparatory step to maintain the quality of anatomical specimens for injection and imaging (Table 3). While basic approaches, such as the use of tap water, are common and effective in clearing blood and clots, certain protocols highlight hyperosmolar solutions flushing for their unique benefits. For example, Frik et al. [16] demonstrated that hyperosmolar solutions effectively displace residual blood and water, reducing the risk of clot formation and tissue swelling during subsequent procedures [16]. Similarly, Meyer et al. [23], emphasized their role in preserving vascular architecture, particularly in embalmed specimens, where these solutions complement chemical preservatives like formaldehyde or Thiel’s solution [16].

**Table 3:** Distribution of flushing methods across the reviewed studies

Flushing method	Number of articles	Percentage of total (%)	Details
Water or Saline (Room temperature)	37	48.05%	Used to remove blood and clots before freezing and fixation. Finishing with an injection of a hyperosmolar solution is sometimes recommended
Formaldehyde solution	16	20.78%	Used diluted. Flushing for ensuring a better preservation
Thiel solution	14	18.18%	Used diluted. Flushing for ensuring a better preservation
No flushing	7	9.09%	Vascular flushing was omitted or not mentioned in these studies
Other methods (e.g., alcohol-based solutions)	4	5.19%	Less common methods used in experimental setups or specific contexts

### Imaging modalities for vascular visualization

The analysis of reviewed studies highlights the predominance of computed tomography (CT) as the most frequently employed radiological imaging modality, accounting for 20 articles (25.6% of the total) (Table 4). This reflects its wide accessibility, high spatial resolution, and capacity to allow detailed vascular reconstructions. For instance, Barry et al. [2] leveraged CT imaging to achieve opacification and 3D reconstruction of coronary arteries in cadaveric hearts, using a combination of 33% iodinated contrast agent and 66% latex under controlled pressure (Fig. 5) [2].

**Table 4:** Imaging modality across the reviewed studies

Imaging modality	Number of articles	Percentage of total (%)
X-Ray	6	7.7%
CT	20	25.6%
MRI	17	21.8%
2 Combined Modalities	4	5.1%
Non-radiological visualization methods	31	39.7%
More than 2 combinations	0	0.00%

Magnetic resonance imaging (MRI) represents 17 studies (21.8%). Despite being less prevalent, MRI demonstrates unique advantages, particularly for soft tissue differentiation and studies involving both vascular and parenchymal structures. Plaisant et al. [27] exemplified this approach by using gadolinium-based contrast agents to delineate vascular details alongside surrounding tissues, underscoring its relevance for combined structural assessments.

Among the 4 studies (5.1%) that utilized dual imaging modalities, namely X-ray, CT and MRI, Renard et al. [30] demonstrated the effectiveness of combining CT and MRI using an optimized injection protocol with colorized latex, gadobutrol, and barium sulfate. This protocol produced high-resolution images of vascular structures and enabled detailed 3D reconstructions, supporting both imaging analysis and subsequent anatomical dissection. A distinct example is shown in Fig. 6. X-ray imaging, while historically significant, accounts for only 6 articles (7.7%), reflecting its limited resolution and reduced utility in contemporary research. 31 included studies (39.7%) did not use radiological imaging. Instead, they relied on macroscopic photographic documentation and microscopic examination after injection to assess vascular filling, anatomical detail, or cast quality. These studies were retained when they provided technical information considered directly relevant to contrast-enhanced vascular injection workflows.

### Casting materials

Concerning casting materials, there is a predominant use of latex, accounting for 59% of the studies, indicating its widespread acceptance (Table 5). Other materials, such as resin and mixed approaches, are less commonly employed, typically in specialized or experimental applications. To date, no study has thoroughly assessed optimal concentrations, viscosities, injection pressures, or the compatibility of different compounds, leaving vascular casting techniques largely reliant on empirical adjustments, which results in variability in opacification, distribution uniformity, and imaging outcomes. The most comprehensive study on the compatibility of different vascular injection materials with CT and MRI imaging was conducted by Plaisant et al. [27], who systematically tested a range of compounds. Their study highlighted the limitations of latex mixed with gadolinium, which generated significant artifacts in MRI imaging, whereas a gelatin-based formulation with gadolinium and minium (red lead) demonstrated optimal results, balancing vascular filling quality, CT radiopacity, and MRI signal compatibility.

**Table 5:** Distribution of casting material used across the reviewed studies

Casting material	Number of articles	Percentage of total (%)
Latex	46	58.97%
Silicone	22	28.21%
Resin	4	5.13%
Other material	3	3.85%
Multiple materials	2	2.56%
Not specified	1	1.28%

### Anatomical regions and vascular systems

Most of the studies are focused on cerebral vasculature, accounting for 35 publications (44.9%). This is followed by studies on the limb vasculature, with 30 articles (38.5%), the heart, with 7 studies (9%). Liver and kidneys, each with 2 studies (2.6%), are less explored.

### Integration of 3D imaging, segmentation, and 3D printing

Reviewed works highlight the synergy of specialized injection protocols and advanced imaging to enhance anatomical understanding or surgical training. Latex-barium sulfate solutions in cerebral venous studies enable the visualization of cerebral venous structures down to 150 μm in diameter by combining high-resolution cone-beam CT imaging with detailed 3D reconstructions of the intracranial vasculature and microsurgical dissection [20]. Epoxy-barium sulfate casting improves visualization of distal arterial branches, achieving high radiopacity and precise 3D CT reconstruction. Complete correlation between imaging and dissection was achieved down to 0.1 mm vessel diameter, and complete coronary opacification through an optimized protocol combining 33% Visipaque ® (iodixanol, GE Healthcare) and 66% latex, injected under controlled pressure of 120 to 150 mmHg [2, 6]. This approach enabled detailed 3D reconstruction of both main and secondary coronary arteries. Although these studies underline the educational and clinical value of such reconstructions, none reported the use of 3D printing to produce physical anatomical models.

### Applications in medical education and surgical training

Postmortem imaging combined with vascular injection techniques has been primarily applied in neurosurgery and peripheral vascular surgery, accounting for 44.9 and 38.5% of the papers, respectively (Table 6). This predominance reflects both the clinical significance of these fields and the relative feasibility of vascular injection techniques in these anatomical regions.

**Table 6:** Distribution of organ studied among the reviewed articles

Organ	Number of articles	Percentage of total (%)
Brain	35	44.87%
Limb Vasculature	30	38.46%
Heart	7	8.97%
Liver	2	2.56%
Kidney	2	2.56%
Other	2	2.56%

Zhao et al. developed a cadaveric model integrating computed tomography with vascular contrast injection for neurosurgical planning [39]. Their protocol, using a 1:8 ratio of contrast agent to silicone rubber, enables uniform vessel opacification, facilitating both high-contrast CT imaging and subsequent anatomical dissection. This model marked a significant step forward by providing both static and dynamic 3D visualizations of cerebrovascular anatomy, allowing for a comprehensive assessment of surgical corridors within the skull base.

More recently Giammattei et al. [17] incorporated CT-based neuronavigation into their cadaveric study to enhance surgical realism in comparing the Combined Petrosal Intertentorial Approach (CPIA) and the Standard Combined Petrosectomy Approach (SCPA). Using five bilaterally dissected specimens, they quantified surgical exposure, angles of approach, and anatomical preservation, leveraging real-time CT data to replicate intraoperative conditions.

Their findings demonstrated that while the SCPA provided greater surgical exposure (18% more skull base area, 17% more brainstem exposure, and 22% greater surgical freedom), it also carried a higher risk of temporal lobe manipulation and venous infarction. In contrast, the CPIA preserved an additional meningeal layer, potentially reducing postoperative complications. By integrating refined vascular injection techniques, CT imaging, and neuronavigation (CT-based image guidance for surgical planning and navigation), this study reinforces the value of high-fidelity cadaveric models for neurosurgical training, bridging the gap between anatomical dissection and live surgery.

Despite these advancements, the use of postmortem vascular imaging remains disproportionately focused on neurosurgery and peripheral vascular applications. While almost 9% of the studies investigated cardiac vasculature, less than 3% examined vascular structures in visceral organs (liver, kidney). This imbalance suggests an underutilized potential for postmortem imaging models in fields such as cardiac and abdominal surgery. Expanding the use of high-resolution vascular injection techniques in these domains could provide valuable insights for surgical training and anatomical research beyond the central nervous and peripheral vascular systems.

### Trends in researcher specializations

The analysis of author qualifications indicates a predominance of surgical and anatomical expertise in the field of intravascular injection techniques. Neurosurgeons (31.4%), anatomists (29.2%), and vascular surgeons (30.2%) collectively account for over 90% of contributing researchers. In contrast, radiologists represent only 2.5%, and other specialties account for 6.6% of total contributions (Table 7).

**Table 7:** Researchers specialization among reviewed articles

Speciality	Percentage of total (%)
Neurosurgeons	31.45%
Anatomists	29.16%
Vascular Surgeons	30.24%
Radiologists	2.54%
Others	6.61%

The underrepresentation of radiologists suggests that the primary focus of these studies remains on anatomical and surgical applications rather than radiological optimization of imaging protocols. This distribution highlights the emphasis on practical applications for surgical planning, anatomical education, and vascular dissection studies rather than imaging methodology development.

Among the reviewed studies, multidisciplinary collaborations involving radiologists were infrequent, and their contributions to imaging protocol optimization and contrast injection standardization were only rarely documented. No standardized imaging protocols were consistently reported across the reviewed literature. To improve the practical usability of this review despite this heterogeneity, representative technical protocols reported in the literature are summarized in Table 8. This table is not intended as a formal guideline, but rather as a structured overview of commonly reported workflows, injectates, imaging options, and their main practical strengths or limitations.

**Table 8:** Representative technical protocols reported for contrast-enhanced vascular injection models used in anatomical teaching and surgical training

Study	Region / model	Specimen / preservation	Preparation and access	Injectate	Imaging	Main practical value	Main limitation
Meyer et al. [23]	Latissimus dorsi / microvascular model	Thiel-embalmed cadavers	Vascular flushing with Ringer lactate (1.5 m hydraulic gradient) before injection	PU4ii elastomer, 30% EMK, 1–2% pigment or 20% Lipiodol, hardener 100:18	Gross dissection, light microscopy, SEM, micro-CT	Easy palpation and dissection of small vessels; compatible with micro-CT and corrosion casting	Specialized material handling; mainly suited to research-oriented microvascular work
Krogager et al. [20]	Cerebral venous anatomy / neurosurgical model	Fresh heads obtained within 48 h; fixation with ethanol + glycerol, then storage in 30% ethanol	Bilateral cervical exposure; saline flushing of ICAs; underwater preparation. Tourniquet used	Latex-iodine mixtures tested (75/50/25% contrast); final protocol: latex–barium (75/25)	Radiographs, cone-beam CT, 3D reconstructions, microsurgical dissection	Direct cross-validation between 3D imaging and dissection in the same specimen; venous filling down to 150 μm; realistic microsurgical handling	Technically demanding; excessive pressure may cause extravasation
Shahbazi et al. [35]	Oral mucosa / maxillofacial flap design	Thiel-embalmed cadavers	Cannulation of CCAs and superior sagittal sinus; 20–30 ml diluted ammonia flush; injection under air pressure	Red latex milk with lead oxide	CT, radiographic visualization, dissection	Clear visualization of vascular pathways and anastomoses for flap/incision planning; good tissue pliability for surgical teaching	Small vessels may remain incompletely filled if clots persist or pressure is insufficient
Bulla et al. [6]	Limbs / distal arterial and perforator anatomy	Fresh cadaveric limb specimens / refrigerated after injection	Humeral or femoral cannulation; warm water flushing until blood cleared; injection monitored by distal leakage	Epoxy resin + barium sulfate + red dye	CT, 3D reconstruction, dissection	Perfect correspondence between CT and dissection; arteries visible down to 0.3 mm on CT and 0.1 mm by direct inspection; hardened vessels facilitate fine dissection	More specific and less flexible than latex-based protocols
Barry et al. [2]	Coronary arteries / heart model	Ex situ formalin-fixed human hearts	Coronary arterial tree rinsed with 60 mL saline; injection pressure 75–120 mmHg	33% iodinated contrast + 66% latex	CT, 3D coronary reconstruction	Detailed opacification of main and secondary coronary branches; strong radiological–anatomical correlation	Slight opacification of small vessels
Renard et al. [30]	Ex vivo anatomical specimens / multimodal model	Fresh ex vivo organs before dissection	Hepatic arterial catheterization (19G), gradual manual injection (20–50 mL); pancreas: portal and arterial dual injection with clamping; flushing not reported	Latex + gadobutrol (3 mL/L) + barite (10%); agar/gelatin, silicone, and resin also tested with multiple CT/MRI contrasts	CT + MRI + gross dissection	Comparative protocol study; identifies the best compromise for combined CT, MRI, and dissection in the same specimen	Iodine, CuSO4, and gadoxetic acid showed inhomogeneous signal or extravasation; agar too soft for dissection; silicone difficult to inject; resin problematic

These protocols do not constitute standardized guidelines, but rather summarize representative workflows, injectates, and imaging strategies reported in the literature

## Discussion

### Main findings and educational relevance

This review highlights the substantial evolution of intravascular injection techniques and associated documentation modalities over the past two decades, from feasibility studies to applications in anatomical education and surgical training. The increasing reliance on high-resolution imaging, particularly CT and MRI, has allowed unprecedented visualization of vascular structures, although methodological limitations continue to hinder reproducibility and standardization.

Although in vivo CTA- and MRA-based morphological studies are more numerous in the broader literature, cadaveric vascular casting models address a different objective: they allow the same specimen to be injected, imaged, and subsequently dissected, thereby supporting direct radiological-anatomical correlation, hands-on anatomical verification, and procedure-oriented surgical training.

**Fig. 2:**
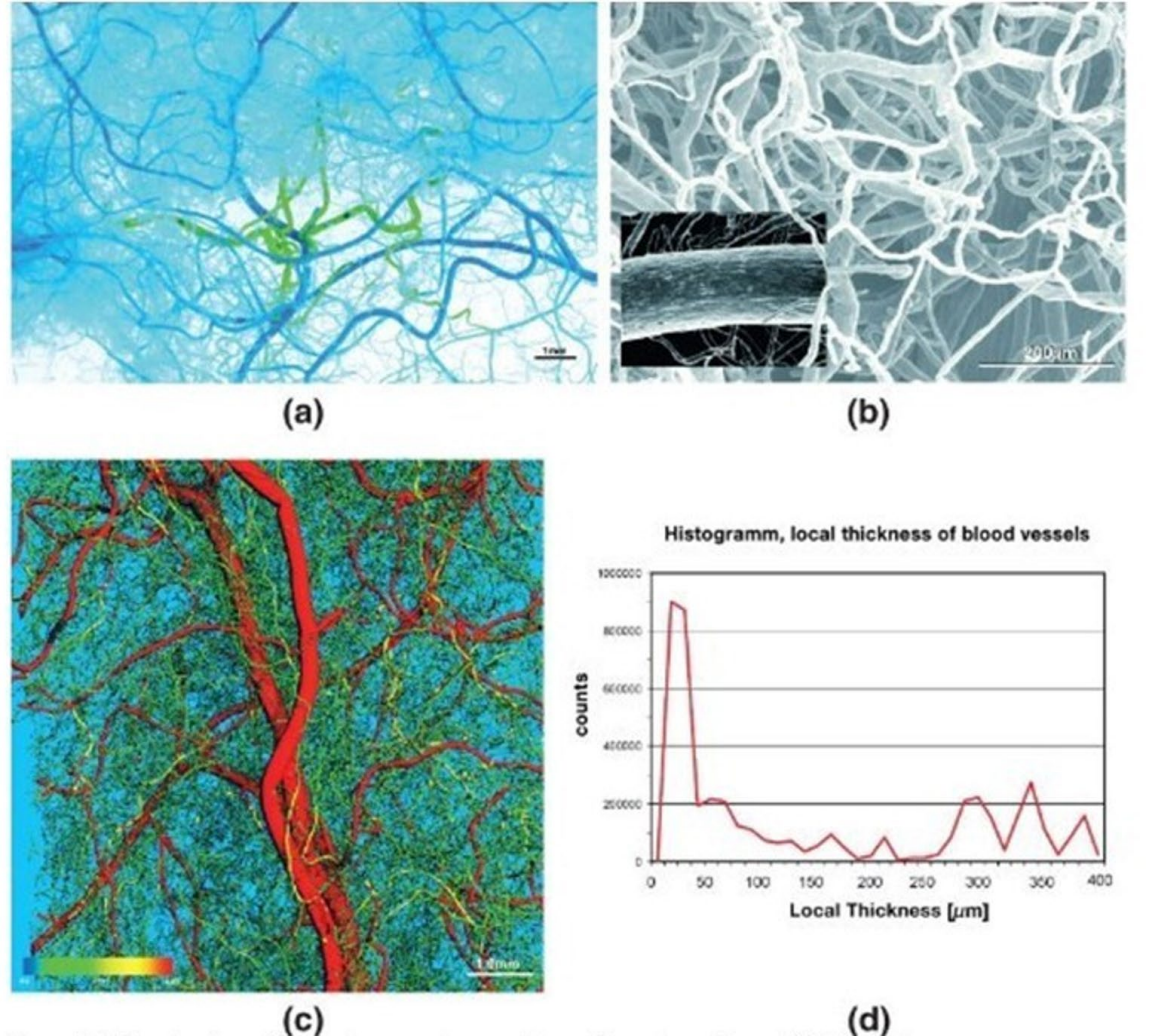
Visualization of vascular corrosion casting using polyurethane (PU4i) elastomer.** a** Macroscopic view of a cast from thoracodorsal and intercostal vessels, revealing dense vascular networks including arteries (blue), intercostal arteries (green), and small distal vessels.** b** SEM image showing the resin microstructure and vessel wall impressions.** c** 3D reconstruction with color-coded vessel diameters derived from micro-CT.** d** Histogram of local vessel thickness derived from image analysis. Adapted with permission from Merver et al. [23]

**Fig. 3:**
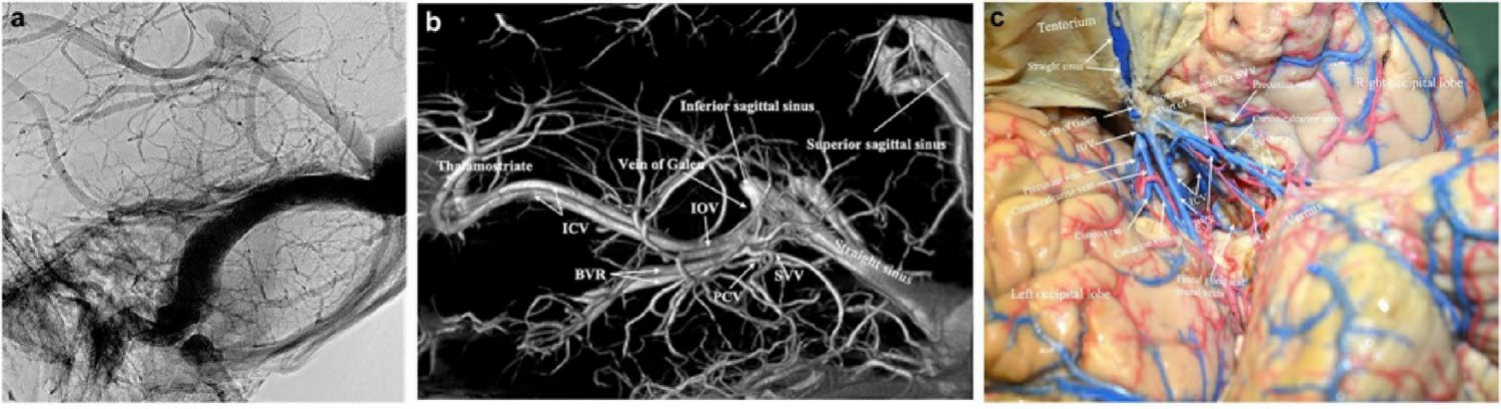
Postmortem visualization of cerebal venous anatomy using latex-barium injection and cone-beam CT.** a** Lateral radiographic projection after injection of a 25% latex-barium mixture into the internal jugular vein, showing the Galenic system and posterior fossa veins.** b** Volume rendering (VRT) from cone-beam CT demonstrating the same venous anatomy is greater detail.** c** Posterior surgical dissection of the same specimen, with arterial injection performed using red latex without barium. Adapted from Krogager et al. [20], licensed under CC BY 4.0

**Fig. 4:**
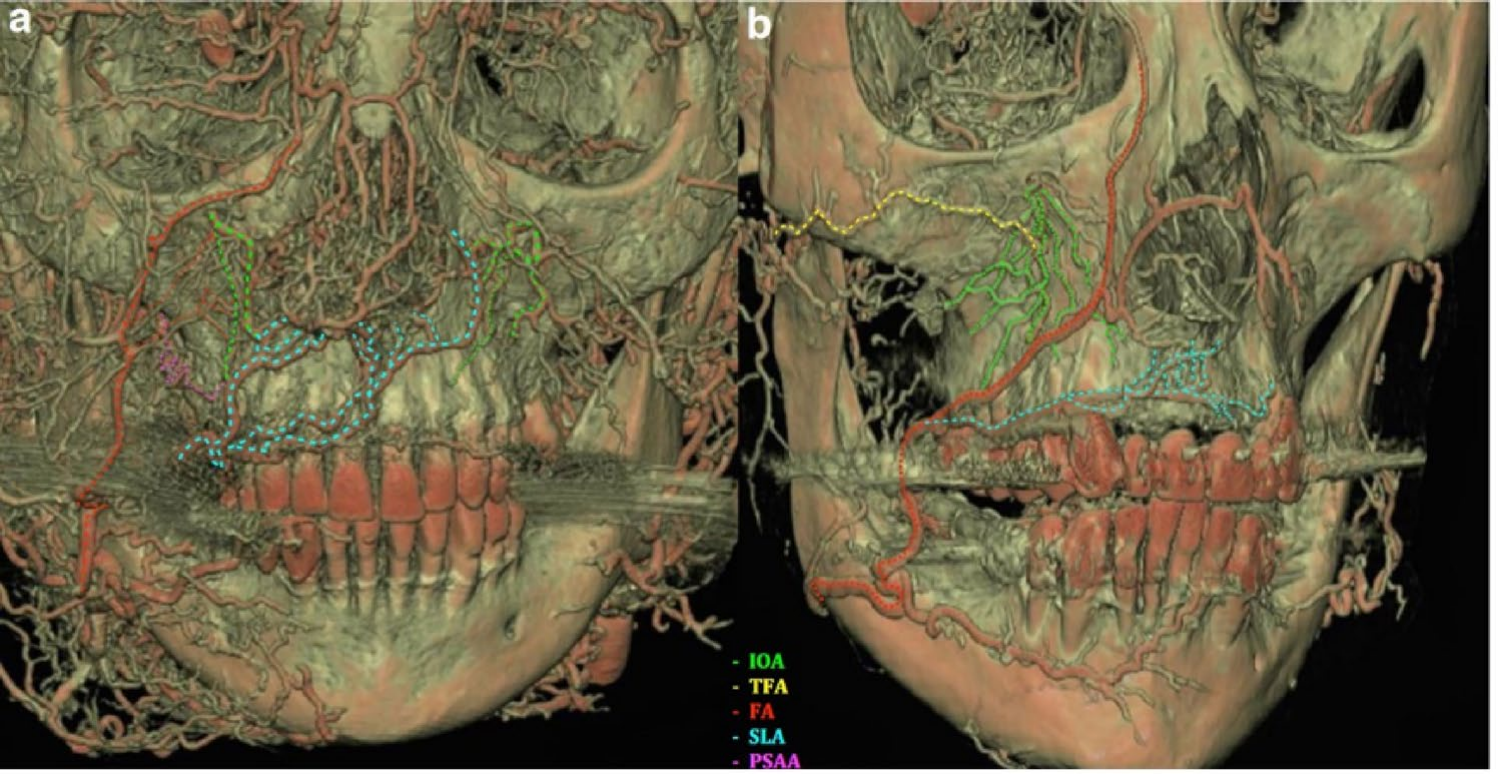
Three-dimensional CT visualization of facial arterial after red latex milk injection.** a** Anterior view showing anastomoses between branches of the facial artery (FA), infraorbital artery (IOA), posterior superior alveolar artery (PSAA), and superior labial artery (SLA). The SLA forms connections with nasal septal and infraorbital branches, including contralateral pathways.** b** Lateral view demonstrating multiple anastomoses among IOA, transverse facial artery (TFA), and FA. Adapted from: Shahbazi et al. [35]. Licensed under CC BY 4.0

**Fig. 5:**
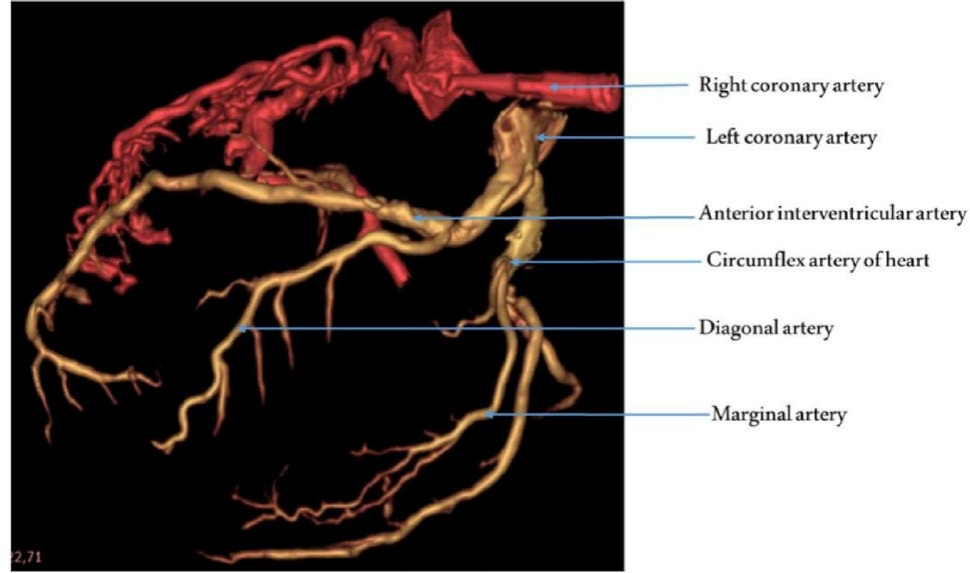
Three-dimensional CT reconstruction of coronary arteries following contrast injection in a human cadaver heart. The image shows clear distinction between the right and left coronary arteries and detailed segmentation of the left coronary artery branches, including the anterior interventricular, circumflex, diagonal, and marginal arteries. Adapetd from Barry et al. [2], with permission

**Fig. 6:**
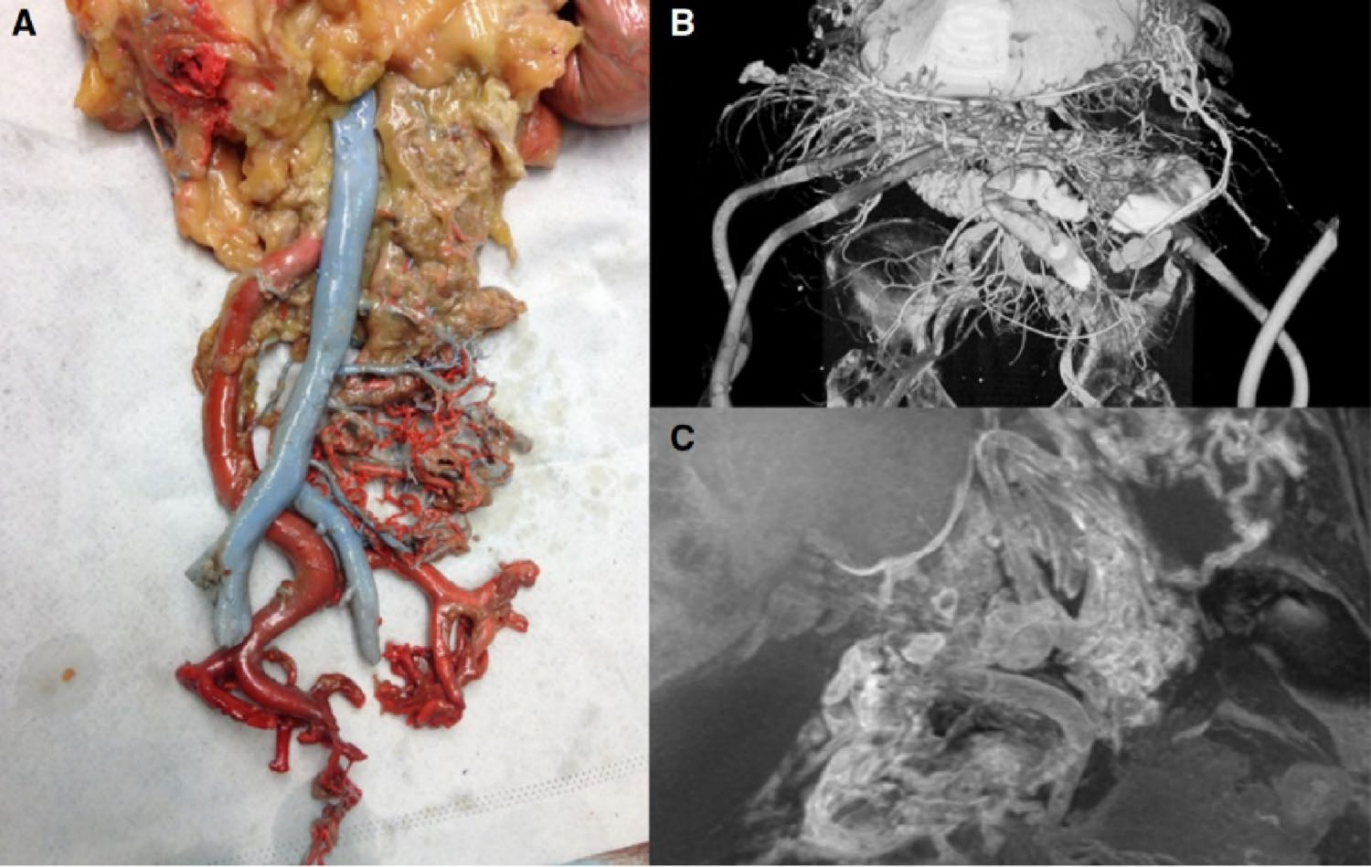
Dissection of the vascular anatomy of the human pancreas following injection of a contrast-enhanced mixture (latex-gadobutrol-barite).** a** Gross anatomical view of the vascular network with arteries labeled in blue and veins in red.** b** Corresponding CT image showing high-resolution visualization of the injected vessels.** c** MRI image of the same region, demonstrating complementary soft tissue contrast and vascular delineation. Adapted from Renard et al. [30]; © 2024 Anatomical Society / Wiley. Reproduced with permission

Beyond these technical considerations, a small number of studies also illustrate the concrete educational and training value of multimodal injected specimens. Although formal educational outcome measures remain scarce in this field, some studies provide concrete evidence of pedagogical value through their methodological design and multimodal integration. Barry et al. [2] demonstrated a precise correspondence between CT imaging and anatomical dissection, with even small-caliber arteries identifiable on both the CT reconstructions and the specimen itself; importantly, the hardened injected vessels also facilitated the identification and dissection of small branches, directly supporting hands-on anatomical training. Krogager et al. [20] extended this principle to neurosurgical anatomy by combining high-resolution cone-beam CT and microsurgical dissection in the same cadaveric heads, allowing direct cross-validation between 3D venous reconstructions and operative anatomy. In that study, venous structures down to 150 μm were visualized, and the preparation retained near-realistic maneuverability, making the model suitable for hands-on neurosurgical training. The studies illustrated in Figs. 2, 3, 4, 5, and 6 further support this pedagogical relevance by showing how injected specimens can be examined across complementary modalities, including gross anatomy, sectional imaging, and three-dimensional reconstruction. Taken together, these examples suggest that the main educational benefit of these models lies less in isolated visualization quality than in their capacity to support spatial understanding, anatomical verification, and procedure-oriented learning through direct radiological-anatomical correlation.

Beyond the descriptive overview provided in Table 8, some practical patterns nevertheless emerge when the reported protocols are interpreted according to their intended use. To make these findings more directly applicable, Table 9 summarizes pragmatic protocol-selection options according to the main objectives encountered in this review, including recurrent anatomy teaching, short-term surgical training, multimodal CT–MRI workflows, and microvascular research. This table is not intended as a formal guideline, but rather as a practical synthesis of reasonable protocol combinations derived from the reviewed literature.

**Table 9:** Practical protocol-selection recommendations according to intended educational, research, and imaging objectives

Primary objective	Typical specimen	Long-term reuse needed	Specimen options / fixation	Common injectate/contrast combinations	Compatible imaging options	Main practical priority	Main caution
Recurrent anatomy teaching	Whole body, large specimen, limb or organ	Yes	Thiel-embalmed or formalin-preserved specimens; fresh specimens unsuitable for repeated long-term use	Latex or silicone + barium or oil-based iodine / hydrophilic iodine; lead oxide in historical or specific protocols	Radiography / fluoroscopy; CT / CBCT	Durability, repeated handling, stable visual contrast	Fixation improves reuse but may reduce vascular permeability, increase leakage risk, and complicate homogeneous filling. Silicone-based mixtures may be more difficult to inject, may require substantial dilution, and should be checked for mixture stability before use
Short-term surgical training	Fresh head, limb, or targeted region	No	Fresh; fresh-frozen; limited-duration unfixed preparations	Latex or silicone-based compounds + barium, hydrophilic or oil-based iodine	Radiography / fluoroscopy; CT / CBCT	Tissue realism, vascular patency, maneuverability	Short usable time; storage constraints. Hydrophilic iodine mixture stability may be less reliable than with barium or oil-based formulations, and phase separation has been reported in some workflows
Multimodal CT–MRI study	Ex situ organ or selected anatomical region	Optional	Fresh ex vivo specimen; carefully prepared preserved specimen in selected workflows	Latex + gadobutrol + barium; agar/gelatin + gadobutrol + barium when gross dissection is not required	CT + MRI	Balanced CT–MRI contrast while preserving sufficient injectate stability for subsequent anatomical assessment	Many multimodal mixtures involve trade-offs: hydrophilic iodine and gadoxetic acid may extravasate, agar/gelatin may be too soft for gross dissection, silicone is difficult to inject, and resin may remain problematic
Microvascular research	Ex situ organ, flap model, or muscle specimen	Optional	Fresh or carefully preserved specimen depending on endpoint	Polyurethane or latex + barium or oil-based iodine; epoxy + barium	Radiography / fluoroscopy; CT / CBCT; micro-CT; microscopy; SEM	Accurate reproduction of distal microvascular architecture for high-resolution imaging, casting, and quantitative analysis	Polyurethane may become rigid, and epoxy is even more brittle; both are well suited to imaging and corrosion casting, but less tolerant of repeated handling

This table proposes reasonable protocol-selection options according to intended use and should not be interpreted as a prescriptive or standardized guideline

### Practical considerations for specimen preparation, casting materials, and imaging compatibility

#### Specimen preparation

The use of whole cadavers or cadaveric parts in anatomical studies encompasses various preservation methods, each suited to different educational, training, and research purposes. Fresh specimens offer the most realistic tissue texture and color, making them ideal for surgical training and detailed anatomical studies, although they must be used promptly to avoid decomposition. Frozen specimens provide a practical alternative, retaining much of the anatomical detail while being easier to handle and store, although freezing can cause tissue artifacts by altering tissue texture and elasticity, which may affect the accuracy of anatomical dissections. Fixed specimens, typically embalmed with formaldehyde or Thiel solutions, offer long-term preservation and safety for extensive educational use, surgical training, and research. Embalming stabilizes tissue structure, allowing repeated dissections and prolonged study periods without significant decomposition [29]. The Thiel method, known for preserving the natural color and consistency of tissues, is particularly valuable for both teaching and surgical practice [38]. Thiel-embalmed specimens, as demonstrated by Shahbazi et al. [35], maintain soft tissue pliability and allow detailed visualization of vascular structures, offering a significant advantage over formalin-fixed specimens, where reduced vascular permeability complicates injection procedures. The ability of Thiel fixation to preserve vascular patency suggests its suitability for imaging-based anatomical studies, particularly when combined with controlled latex injection. However, chemical preservation alters the physical properties of tissues, thereby affecting the realism of the dissecting experience.

One of the critical challenges in using fixed organs for anatomical studies is ensuring effective perfusion and permeability of the vascular bed. While fixation techniques offer excellent preservation and durability, they may alter the natural permeability of vascular structures, which may limit the effectiveness of intravascular injection techniques and compromise the ability of the vascular bed to adequately receive and distribute injected substances. Formalin fixation is known to alter the biomechanical properties of soft tissues by cross-linking proteoglycan monomers, leading to protein denaturation [1]. This process causes blood clotting and results in tissues that are stiff, rigid, brittle, and more difficult to handle during dissection. Despite this concern, the current state of research lacks comprehensive studies specifically investigating the permeability of fixed vascular beds. As a result, there is a significant knowledge gap regarding the extent to which fixation affects the anatomical accuracy and educational value of these models.

Each preservation modality, whether refrigeration, freezing, or fixation, has its own advantages and disadvantages, making it essential to choose the most appropriate method according to the specific goals of anatomical education, surgical training, or research. Effective specimen preparation is critical for successful intravascular injection and includes appropriate selection and preservation of cadavers, identification of vascular access points, and preparation of injection solutions.

#### Injection techniques

Among the preparatory steps that influence the success of intravascular injection and imaging, vascular flushing is often emphasized as a crucial process. This procedure involves clearing the vascular system of blood and clots, which can otherwise obstruct the pathways and hinder the effectiveness of injections [24]. For fresh specimens, flushing is typically performed with tap water or saline solutions immediately postmortem in order to maintain clear and patent vessels. Frozen specimens also benefit from pre- or post-freezing flushing to remove clots, while fixed specimens, particularly those treated with formaldehyde or Thiel solutions [26], undergo flushing before fixation to ensure thorough penetration of the fixative. However, despite its importance, vascular flushing is omitted in some protocols, indicating variability in preparatory practices. Studies have reported benefits of hyperosmolar flushing solutions for reducing swelling, clot formation and improving vascular patency [16], yet no comparative studies have established optimal osmolarity thresholds or solution compositions. This has led to inconsistent methodologies across studies, limiting reproducibility.

Colored latex or other casting materials are typically injected into the arterial system via cannulation, often through a major artery such as the femoral or carotid artery, or through a more distal vessel depending on the targeted region. When isolated limbs or organs are injected, local access is preferred to reduce material consumption and improve precision. Injection can be performed using syringes, peristaltic pumps, or gravity-based systems. For instance, Doomernik et al. [11] used a gravity infusion setup with a constant pressure equivalent to a 2-m fluid column to ensure steady flow, together with backflow control and clamping of collateral vessels. Injection was continued until flow cessation and stabilization of the fluid level confirmed complete filling. Pressure monitoring is occasionally employed to optimize perfusion and avoid overdistension. Meticulous handling is required to prevent air bubbles, vessel rupture, and incomplete distribution.

Another critical aspect is the role of injection parameters in determining vascular opacification. The reviewed studies employ varied injection pressures and techniques, yet the impact of these factors on contrast distribution remains insufficiently documented. Standardizing pressure thresholds and comparing manual versus controlled pump-assisted injections would provide greater consistency in anatomical visualization. Future studies should aim to establish optimized protocols tailored to different preservation methods, ensuring reproducibility and comparability across institutions.

#### Radiological contrast media injection

To enable imaging, radiological contrast agents such as barium sulfate or iodine-based compounds for CT, and gadolinium-based agents for MRI, can be integrated into the injection process. Two main approaches are reported. In the simultaneous method, the contrast agent is mixed directly with the casting material, ensuring co-distribution and simplifying the procedure, but the high viscosity may limit perfusion [30]. In the sequential method, the contrast agent is injected first, allowing better vascular penetration because of its lower viscosity, although it requires careful timing to avoid displacement of the contrast medium during the casting phase [18, 27].

#### Applications in anatomical education

The incorporation of radiological contrast media into casting materials is essential for visualizing vascular structures using imaging modalities such as CT and MRI. Without these contrast agents, vessels sometimes remain indistinguishable from the surrounding tissues. This technique allows detailed imaging of the vasculature, providing valuable resources for anatomical education and research. Moreover, imaging of the injected vessels corresponds precisely to the molding on the physical specimen, allowing direct correlation between radiological images and anatomical structures.

In anatomical education, three-dimensional models derived from imaging studies are used to improve spatial comprehension of anatomical structures [3, 5, 15]. These models are employed in a multimodal approach, combining physical specimens, radiological imaging, and 3D reconstructions to provide comprehensive anatomical insights. This integration allows direct comparison between dissected specimens and their corresponding radiological images, while 3D models offer additional perspectives and detailed visualization of complex structures. To complement these methods, digital technologies such as VR and AR are sometimes used. These tools create immersive learning environments that enable students to explore anatomical structures interactively, adding depth to the educational experience [7, 10].

The dominance of computed tomography (CT) in intravascular injection studies reflects its accessibility, high spatial resolution, and ease of integration with contrast-enhanced vascular reconstructions. Its limited soft tissue contrast remains a drawback when simultaneous visualization of vascular and parenchymal structures is required. Magnetic resonance imaging (MRI), while offering superior soft tissue differentiation, is used less frequently despite its clear advantages in postmortem anatomical studies. The discrepancy in usage does not appear to stem from scientific limitations but rather from practical constraints: MRI is more expensive, requires longer acquisition times, and is often less accessible due to high clinical demand. These factors are rarely explicitly addressed in the reviewed studies, suggesting that decisions regarding imaging modality selection may be influenced more by logistical feasibility than by comparative technical evaluation. Additionally, the near absence of comparative studies evaluating multimodal imaging approaches limits current understanding of how combined imaging strategies might enhance vascular visualization. The shift away from conventional X-ray methods underscores the preference for volumetric imaging, yet the full potential of hybrid imaging, such as combining CT with MRI or leveraging advanced post-processing techniques, remains underexplored. Future research should prioritize systematic comparisons of single- versus multi-modality approaches, not only to refine imaging protocols but also to establish objective criteria for selecting the most appropriate modality based on specific anatomical structures or to meet other procedural requirements.

#### Casting materials for intravascular injections

Latex is a widely used material because of its flexibility, ease of use, and cost-effectiveness. It allows thorough filling of vessels, making it particularly suitable for creating detailed anatomical models, as demonstrated in studies focusing on neurovascular anatomy and the vascular systems of the extremities [9, 14]. Latex, typically stabilized with ammonia to prevent vulcanization, can be diluted with water to reduce its viscosity, which extends curing time but facilitates precise injections in very small and complex vascular networks [36]. However, latex tends to degrade over time, which limits its utility for long-term educational purposes, particularly when compared with silicone- or resin-based alternatives. Silicone is another popular choice, valued for its durability and flexibility, but it can be expensive and has a naturally higher viscosity than latex, which can make the injection process challenging [36]. To address this, silicone is often diluted by up to 30% using specific fluids designed to lower its viscosity, thereby facilitating manual injection. It is important to note that diluting silicone reduces its tensile strength after hardening, meaning that it becomes less resistant to stretching or deformation once set. Despite these challenges, silicone remains the preferred material for creating long-lasting anatomical models [19].

The choice of casting material significantly influences vascular opacification and imaging quality. Latex remains the most commonly used compound, yet its interaction with imaging modalities is not always optimal, particularly in MRI, where it may generate artifacts when mixed with gadolinium-based agents. Alternatives, such as gelatin-based compounds enriched with contrast media, have demonstrated superior compatibility across both CT and MRI, enabling clearer vascular delineation. However, the lack of standardized protocols for viscosity, injection pressure, and radiopacity calibration leads to high variability between studies. Addressing these methodological inconsistencies through comparative evaluations of casting materials could improve the reproducibility and anatomical accuracy of vascular imaging.

Resins such as polyurethane and epoxy are also used in vascular casting. Polyurethane hardens quickly and is resistant to acids, making it suitable for specific techniques such as injection-corrosion (Fig. 2) [23]. However, mixing resins with certain contrast agents, such as iodine or barium sulfate, can lead to emulsion reactions, which may prevent their use in vascular imaging. Epoxy is not prone to these reactions, but it requires degassing, that is, removal of air bubbles trapped during mixing, to ensure a smooth and uniform cast [11]. This process, together with its toxicity, necessitates the use of specialized equipment.

Gelatin and agar are often used when a softer, more flexible model is needed. These materials are easy to handle and can be mixed with a variety of contrast agents, including barium sulfate, lead oxide, and gadolinium. This makes them versatile for different types of imaging studies. These mixtures must be warmed immediately before injection to prevent premature hardening. However, when they are injected into specimens that are often preserved by refrigeration, the low temperature causes the mixture to cool rapidly and harden, thereby complicating the procedure [11]. In conclusion, the selection of casting material should align with the specific goals of the anatomical study.

#### Properties and selection of radiological contrast media

The use of radiological contrast media in anatomical studies has evolved significantly alongside advances in medical imaging techniques such as CT and MRI. Initially, contrast agents such as barium sulfate, combined with hardening materials and dyes, were used to enhance vascular visibility in postmortem specimens [32, 33]. Barium sulfate, often mixed with stabilizers [12], was one of the earliest contrast agents used in anatomical studies. This mixture provided adequate radiopacity, making it suitable for standard X-ray imaging while maintaining the structural integrity of the vasculature during dissection.

As imaging techniques advanced, iodine-based contrast agents, widely used in clinical practice, became a standard for in vivo imaging because of their excellent radiopacity and ease of injection [18]. Nevertheless, their application in postmortem studies is limited because they tend to diffuse rapidly through vessel walls when dissolved in water, providing only moderate and potentially inaccurate radiopacity [18]. Consequently, lead oxide, despite its toxicity, became a preferred choice for vascular visualization [34], particularly when mixed with silicone, latex, or gelatin for microangiography. Although effective, the high toxicity of lead oxide restricts its routine use in laboratories without specialized equipment.

Barium sulfate re-emerged as a safer alternative to lead oxide [12], particularly for high-resolution micro-CT scans, owing to its non-toxicity and improved radiopacity in certain solvent mixtures. In contrast, gadolinium-based agents, favored for MRI studies because of their high T1-relaxivity and stability [22], offer another option. These agents, especially when mixed with gelatin, prevent extravasation, making them ideal for detailed imaging studies [18]. However, without the inclusion of radiopaque agents, such mixtures do not permit simultaneous CT imaging.

The development of specialized casting materials, such as those used in microangiography, has further refined the application of contrast media in anatomical studies [21]. Although costly, these materials offer enhanced imaging capabilities and the potential for detailed anatomical analysis without the risks associated with more traditional substances. For instance, Krogager et al. [20] used latex-barium sulfate injections combined with cone-beam CT imaging to visualize cerebral venous structures as small as 150 μm. Volumetric reconstructions of the Galenic and posterior fossa venous systems were clearly visible on both radiographic projection and 3D volume rendering. These imaging results (Fig. 3) were directly correlated with subsequent microsurgical dissection under operating microscope magnification using fine micro-instruments, highlighting the precision and anatomical fidelity achievable when combining vascular casting, advanced imaging, and targeted dissection. Similarly, Bulla et al. [6] employed an epoxy-barium sulfate mixture to achieve high-resolution imaging of peripheral arteries, allowing 3D CT reconstructions with visualization down to 0.1 mm vessel diameter. Combining these advanced contrast agents and casting materials with modern imaging technologies thus allows comprehensive anatomical studies that integrate imaging and dissection, bridging the gap between anatomy and radiology in educational settings.

This variability contributes to heterogeneity in opacification quality and anatomical fidelity. Rather than supporting formal consensus guidelines at this stage, the currently available literature mainly allows the identification of practical methodological considerations, including the importance of controlled pressure settings, appropriate agent concentrations, and careful flushing strategies. Further comparative studies would be valuable to clarify which combinations are the most robust across different anatomical contexts and preservation methods.

### Interdisciplinary perspective and the role of radiologists

One of the most striking findings is the underrepresentation of radiologists among contributing authors. Neurosurgeons (31.4%), anatomists (29.2%), and vascular surgeons (30.2%) dominate the field, while radiologists account for only 2.5% of contributors. This lack of radiological expertise may contribute to the absence of standardized imaging protocols and contrast agent optimization.

The present review was conducted from complementary expertise in anatomy, forensic medicine, and cadaveric imaging, including postmortem angiography, rather than from a radiology-led perspective. This positioning may help explain the particular emphasis placed on specimen preparation, radiological-anatomical correlation, and the practical educational use of injected cadaveric models.

Radiologists are uniquely positioned to refine imaging acquisition parameters, assess contrast media distribution kinetics, and ensure consistency in imaging methodologies. Greater interdisciplinary collaboration between anatomical researchers, surgeons, and radiologists could improve protocol standardization and imaging reproducibility, particularly for multi-modal studies combining CT, MRI, and fluoroscopic techniques.

### Priorities for future research

To advance the field, several priorities should be addressed:

Comparative refinement of injection techniques: Further comparative studies should help clarify the most robust combinations of contrast agent, injection volume or pressure, and flushing protocols according to anatomical region, preservation method, and intended educational or research use.

Comparative studies on imaging modalities. Systematic investigations comparing CT, MRI, and hybrid imaging techniques could help determine the most appropriate modality or combination of modalities based on specific objectives. The integration of radiological imaging with digital microscopy, 3D surface scanning, photogrammetry, corrosion casting techniques, and advanced manufacturing methods such as 3D printing and even tissue bioprinting is expected to play an increasing role in future research.

Digital integration. Integration into digital teaching environments such as DICOM viewers, or immersive tools like virtual and augmented reality, may further enhance the pedagogical utility of these datasets.

Future research should focus on ensuring the compatibility of casting materials, preservation methods, and imaging modalities, in order to enable their combined use and full exploitation.

### Limitations

This review has several limitations. First, the included studies show marked heterogeneity in protocols, including differences in injection materials, concentrations, pressures, and imaging settings, which limits direct comparison. Reporting quality also varies widely, and many articles provide incomplete methodological details, reducing the accuracy of cross-study interpretation. A language bias cannot be excluded, as the search relied primarily on English terms and may have missed relevant non-English publications. In addition, publication bias is likely, since unsuccessful or suboptimal injections are rarely reported. The literature also combines cadaveric conditions that differ substantially (fresh, embalmed, Thiel), making it difficult to generalize findings. Because only a small number of studies provide direct head-to-head comparisons between products, pressures, or modalities, quantitative synthesis or meta-analysis was not feasible. In addition, titles, abstracts, full texts, and data extraction were handled by a single reviewer, which may have introduced selection bias or extraction bias despite repeated verification for consistency. Finally, many studies use different anatomical endpoints, validation approaches, or definitions of “successful opacification,” further limiting the ability to derive broadly applicable methodological recommendations.

## Conclusion

Vascular injection techniques, whether documented by radiological imaging or by other post-injection assessment methods, continue to evolve, and their contributions to anatomy teaching and surgical training are increasingly recognized. While CT remains the most widely used modality, MRI and hybrid approaches hold significant potential. The marked variability in casting materials, injection parameters, and imaging protocols underscores the need for clear and well-documented methodological references. Rather than providing standardized guidelines, the present review combines a structured overview of representative protocols reported in the literature with a pragmatic protocol-selection framework according to intended use. By consolidating evidence across imaging methods, injectates, anatomical applications, and selected technically relevant non-radiological workflows, this synthesis aims to provide a more directly applicable reference for teams implementing or refining vascular injection models in anatomy teaching, surgical training, and research.

## Supplementary Information

Below is the link to the electronic supplementary material.

Supplementary Material 1

## Data Availability

No new datasets were generated. All data extracted from the included studies are contained in the manuscript and its supplementary material.
