# Supplementary material for: Intravascular injection of colored molding materials for human anatomy teaching: a literature review
Source: Surg Radiol Anat. 2026 Jul 14;48(1):177. doi: 10.1007/s00276-026-03938-3 (PMC13369457; doi:10.1007/s00276-026-03938-3)
Supplement: Supplementary file 1 — Supplementary Material 1 [file 276_2026_3938_MOESM1_ESM.pdf]

Online Resource 1. Full list of studies included in the review

Article title: Intravascular injection of colored molding materials for human anatomy teaching: a literature review

Journal: Surgical and Radiologic Anatomy

Authors: Sami Schranz, Hugues Cadas, Sara Sabatasso

Affiliations: Faculty Unit of Anatomy and Morphology (UFAM), University Center of Legal Medicine Lausanne-Geneva (CURML), Lausanne University Hospital and University of Lausanne, Lausanne, Switzerland; Faculty of Biology and Medicine, University of Lausanne, Lausanne, Switzerland

Corresponding author e-mail: sami.schranz@unil.ch

No.	Study	Full reference
1	Alvernia et al. (2010)	Alvernia JE, Pradilla G, Mertens P, et al (2010) Latex Injection of Cadaver Heads: Technical Note. Operative Neurosurgery 67:ons362–ons367. https://doi.org/10.1227/NEU.0b013e3181f8c247
2	An et al. (2012)	An X, Yue B, Lee J-H, et al (2012) Arterial anatomy of the gracilis muscle as determined by latex injection and glycerin transparency. Clin Anat 25:231–234. https://doi.org/10.1002/ca.21217
3	Arnold et al. (1991)	Arnold MM, Kreel L, Lo YF, Law H (1991) Are the hepatic arteries “end arteries”? Invest Radiol 26:337–342. https://doi.org/10.1097/00004424-199104000-00010
4	Athlani et al. (2021)	Athlani L, Sapa M-C, De Almeida Y-K, et al (2021) A new capsulotomy-based dorsal approach to the wrist: A cadaver study. Hand Surg Rehabil 40:134–138. https://doi.org/10.1016/j.hansur.2020.10.015
5	Baena et al. (2019)	Baena Pinilla S, Cisneros Gimeno AI, Obón Nogués J, et al (2019) An innovative quick solidifying technique for the forensic investigation of brain circulation using addition silicones. Forensic Science International 298:398–401. https://doi.org/10.1016/j.forsciint.2019.02.057
6	Baidya et al. (2013)	Baidya NB, Tang C-T, Ammirati M (2013) Intradural endoscope-assisted anterior clinoidectomy: a cadaveric study. Clin Neurol Neurosurg 115:170–174. https://doi.org/10.1016/j.clineuro.2012.05.003
7	Baker (1999)	Baker J. (1999) COR-TECH PR-10 Silicone: Initial Trials in Plastinating Human Tissue. J Int Soc Plast 14:13–19. https://doi.org/10.56507/XVUK7879
8	Barry et al. (2024)	Barry M, Gun M, Chabry Y, et al (2024) Optimizing coronary artery opacification and 3D reconstruction from human cadaver hearts in anatomy research. Curr Probl Cardiol 49:102216. https://doi.org/10.1016/j.cpcardiol.2023.102216
9	Bergeron et al. (2007)	Bergeron L, Tang M, Morris SF (2007) The Anatomical Basis of the Deep Circumflex Iliac Artery Perforator Flap with Iliac Crest. Plastic and Reconstructive Surgery 120:252. https://doi.org/10.1097/01.prs.0000264392.42396.a3
10	Bulla et al. (2014)	Bulla A, Casoli C, Farace F, et al (2014) A new contrast agent for radiological and dissection studies of the arterial network of anatomic specimens. Surg Radiol Anat 36:79–83. https://doi.org/10.1007/s00276-013-1143-z
11	Carr et al. (2018)	Carr LW, Brooke SM, Johnson TS, Michelotti BF (2018) Reexploring the Anatomy of the Distal Humerus for its Role in Providing Vascularized Bone. Plast Reconstr Surg Glob Open 6:e1636. https://doi.org/10.1097/GOX.0000000000001636
12	Chen et al. (1998)	Chen Y-G, Cook PA, McClinton MA, et al (1998) Microarterial anatomy of the lesser toe proximal interphalangeal joints. The Journal of Hand Surgery 23:256–260. https://doi.org/10.1016/S0363-5023(98)80123-8
13	Cho et al. (2017)	Cho JH, Kim JW, Park HW, et al (2017) Arterial supply of the human soft palate. Surg Radiol Anat 39:731–734. https://doi.org/10.1007/s00276-016-1798-3
14	Cilliers and Page (2016)	Cilliers K, Page BJ (2016) Detailed description of the anterior cerebral artery anomalies observed in a cadaver population. Annals of Anatomy - Anatomischer Anzeiger 208:1–8. https://doi.org/10.1016/j.aanat.2016.04.036
15	Çırak et al. (2020)	Çırak M, Yağmurlu K, Soldozy S, et al (2020) Common Challenges and Solutions Associated with the Preparation of Silicone-Injected Human Head and Neck Vessels for Anatomical Study. Brain Sciences 11:32. https://doi.org/10.3390/brainsci11010032
16	Crosthwaite et al. (1987)	Crosthwaite GL, Taylor GI, Palmer JH (1987) A new radio-opaque injection technique for tissue preservation. Br J Plast Surg 40:497–501. https://doi.org/10.1016/s0007-1226(87)90080-4

No.	Study	Full reference
17	Dashner et al. (2005)	Dashner RA, Clark DL, Kangarlu A, et al (2005) Epoxy-resin injection of the cerebral arterial microvasculature: An evaluation of the limits of spatial resolution in 8 Tesla MRI. Clin Anat 18:164–170. https://doi.org/10.1002/ca.20070
18	De la Garza et al. (1992)	de la Garza O, Lierse W, Steiner D (1992) Anatomical study of the blood supply in the human shoulder region. Acta Anat (Basel) 145:412–415. https://doi.org/10.1159/000147399
19	De Bournonville et al. (2019)	de Bournonville, Sébastien, Vangrunderbeeck, Sarah, Kerckhofs, Greet, Contrast-Enhanced MicroCT for Virtual 3D Anatomical Pathology of Biological Tissues: A Literature Review, Contrast Media & Molecular Imaging, 2019, 8617406, 9 pages, 2019. https://doi.org/10.1155/2019/8617406
20	Demondion et al. (2000)	Demondion X, Delfaut EM, Drizenko A, et al (2000) Radio-anatomic demonstration of the vertebral lumbar venous plexuses: an MRI experimental study. Surg Radiol Anat 22:151–156. https://doi.org/10.1007/s00276-000-0151-y
21	Ding et al. (2008)	Ding H-M, Yin Z-X, Zhou X-B, et al (2008) Three-dimensional visualization of pelvic vascularity. Surg Radiol Anat 30:437–442. https://doi.org/10.1007/s00276-008-0348-z
22	Donato et al. (2007)	Donato P, Coelho P, Rodrigues H, et al (2007) Normal vascular and biliary hepatic anatomy: 3D demonstration by multidetector CT. Surg Radiol Anat 29:575–582. https://doi.org/10.1007/s00276-007-0233-1
23	Doomernik et al. (2016)	Doomernik DE, Kruse RR, Reijnen MMPJ, et al (2016) A comparative study of vascular injection fluids in fresh-frozen and embalmed human cadaver forearms. J Anat 229:582–590. https://doi.org/10.1111/joa.12504
24	Durongphan et al. (2022)	Durongphan A, Suksantilap S, Panrong N, et al (2022) Latex-injected, non-decapitated, saturated salt method-embalmed cadaver technique development and application as a head and neck surgery training model. PLoS ONE 17:e0262415. https://doi.org/10.1371/journal.pone.0262415
25	Edizer et al. (2003)	Edizer M, Mağden O, Tayfur V, et al (2003) Arterial anatomy of the lower lip: a cadaveric study. Plast Reconstr Surg 111:2176–2181. https://doi.org/10.1097/01.PRS.0000060110.18366.49
26	Effendi et al. (2014)	Effendi ST, Rao VY, Momin EN, et al (2014) The 1-piece transbasal approach: operative technique and anatomical study. J Neurosurg 121:1446–1452. https://doi.org/10.3171/2014.8.JNS132609
27	Erdogmus and Govsa (2008)	Erdogmus S, Govsa F (2008) Anatomic characteristics of the ophthalmic and posterior ciliary arteries. J Neuroophthalmol 28:320–324. https://doi.org/10.1097/WNO.0b013e318183c4bb
28	Fang et al. (2019)	Fang Y, Wu H, Zhao W, Cheng L (2019) Endoscopic transvestibular anatomy of the infratemporal fossa and upper parapharyngeal spaces for clinical surgery: a cadaver study. Eur Arch Otorhinolaryngol 276:1799–1807. https://doi.org/10.1007/s00405-019-05410-y
29	Garcia-Gonzalez et al. (2012)	Garcia-Gonzalez U, Cavalcanti DD, Agrawal A, et al (2012) Anatomical study on the “perforator-free zone”: reconsidering the proximal superior cerebellar artery and basilar artery perforators. Neurosurgery 70:764–772; discussion 771-772. https://doi.org/10.1227/NEU.0b013e3182351f8e
30	Gargollo et al. (2021)	Gargollo PC, Ahmed ME, Prieto M, et al (2021) Feasibility Study of Vascularized Composite Urinary Bladder Allograft Transplantation in a Cadaver Model. J Urol 206:115–123. https://doi.org/10.1097/JU.0000000000001699
31	Genain et al. (2018)	Genain M-A, Morlet A, Herrtage M, et al (2018) Comparative anatomy and angiography of the cardiac coronary venous system in four species: human, ovine, porcine, and canine. J Vet Cardiol 20:33–44. https://doi.org/10.1016/j.jvc.2017.10.004
32	Goyal et al. (2014)	Goyal N, Setabutr D, Goldenberg D (2014) Transoral robotic study of the vascular anatomy of the head and neck. J Robot Surg 8:57–61. https://doi.org/10.1007/s11701-013-0427-y
33	Grose et al. (2008)	Grose AW, Gardner MJ, Sussmann PS, et al (2008) The surgical anatomy of the blood supply to the femoral head: description of the anastomosis between the medial femoral circumflex and inferior gluteal arteries at the hip. J Bone Joint Surg Br 90:1298–1303. https://doi.org/10.1302/0301-620X.90B10.20983
34	Heymans et al. (2004)	Heymans O, Nélissen X, Peters S, et al (2004) New Approach to Vascular Injection in Fresh Cadaver Dissection. J reconstr Microsurg 20:311–315. https://doi.org/10.1055/s-2004-824889

No.	Study	Full reference
35	Ilgaz et al. (2024)	Ilgaz HB, Urgun K, Yener U, et al (2024) Microsurgical anatomy of the olfactory filaments in the nasal mucosa. J Neurosurg 141:555–563. https://doi.org/10.3171/2024.1.JNS23920
36	Kakou et al. (2000)	Kakou M, Destrieux C, Velut S (2000) Microanatomy of the pericallosal arterial complex. J Neurosurg 93:667–675. https://doi.org/10.3171/jns.2000.93.4.0667
37	Kalhor et al. (2009)	Kalhor M, Beck M, Huff TW, Ganz R (2009) Capsular and pericapsular contributions to acetabular and femoral head perfusion. J Bone Joint Surg Am 91:409–418. https://doi.org/10.2106/JBJS.G.01679
38	Kaya et al. (2006)	Kaya AH, Sam B, Celik F, Türe U (2006) A quick-solidifying, coloured silicone mixture for injecting into brains for autopsy: technical report. Neurosurg Rev 29:322–326; discusson 326. https://doi.org/10.1007/s10143-006-0032-x
39	Kilinc et al. (2021)	Kilinc MC, Basak H, Çoruh AG, et al (2021) Endoscopic Anatomy and a Safe Surgical Corridor to the Anterior Skull Base. World Neurosurg 145:e83–e89. https://doi.org/10.1016/j.wneu.2020.09.106
40	Kostopoulos et al. (2006)	Kostopoulos E, Casoli V, Verolino P, Papadopoulos O (2006) Arterial blood supply of the extensor apparatus of the long fingers. Plast Reconstr Surg 117:2310–2318; discussion 2319. https://doi.org/10.1097/01.prs.0000218799.33322.7f
41	Koutsarnakis et al. (2016)	Koutsarnakis C, Liakos F, Liouta E, et al (2016) The cerebral isthmus: fiber tract anatomy, functional significance, and surgical considerations. J Neurosurg 124:450–462. https://doi.org/10.3171/2015.3.JNS142680
42	Krogager et al. (2023)	Krogager ME, Dahl RH, Poulsgaard L, et al (2023) Combined cone-beam CT imaging and microsurgical dissection of cadaver specimens to study cerebral venous anatomy: a technical note. Surg Radiol Anat 45:1177–1184. https://doi.org/10.1007/s00276-023-03195-8
43	Kuzucu et al. (2023)	Kuzucu P, Çeltikçi P, Demirtaş OK, et al (2023) Arterial Supply of the Basal Ganglia: A Fiber Dissection Study. Oper Neurosurg (Hagerstown) 24:e351–e359. https://doi.org/10.1227/ons.0000000000000612
44	Kwiatkowska and Ciszek (2000)	Kwiatkowska M, Ciszek B (2000) The anatomy of the median branches of the basilar artery. Folia Morphol (Warsz) 59:323–325
45	Lopez et al. (2008)	Lopez R, Lauwers F, Paoli JR, et al (2008) The vascular system of the upper eyelid. Anatomical study and clinical interest. Surg Radiol Anat 30:265–269. https://doi.org/10.1007/s00276-008-0323-8
46	Marino et al. (2024)	Marino S, Dannhoff G, Destrieux C, Maldonado IL (2024) Frontal trans opercular approaches to the insula: building the mental picture from procedure-guided anatomical dissection. Surg Radiol Anat 46:1331–1344. https://doi.org/10.1007/s00276-024-03409-7
47	Martins and Martins (2020)	Martins AC de A, Martins C (2020) Surgical anatomy of caudate bile ducts: Silicon-injected cadaveric-livers dissected under magnification. Ann Hepatobiliary Pancreat Surg 24:415–420. https://doi.org/10.14701/ahbps.2020.24.4.415
48	Meyer et al. (2007)	Meyer EP, Beer GM, Lang A, et al (2007) Polyurethane elastomer: A new material for the visualization of cadaveric blood vessels. Clinical Anatomy 20:448–454. https://doi.org/10.1002/ca.20403
49	Nicol et al. (2019)	Nicol P, Constans J-M, d'Ortho M-P, et al (2019) The parapharyngeal adipose corpus: anatomic and radiologic study. Surg Radiol Anat 41:809–813. https://doi.org/10.1007/s00276-019-02230-x
50	Nikolaou et al. (2004)	Nikolaou K, Becker CR, Muders M, et al (2004) Multidetector-row computed tomography and magnetic resonance imaging of atherosclerotic lesions in human ex vivo coronary arteries. Atherosclerosis 174:243–252. https://doi.org/10.1016/j.atherosclerosis.2004.01.041
51	Oberlin et al. (1992)	Oberlin C, Salon A, Pigeau I, et al (1992) Three-dimensional reconstruction of the carpus and its vasculature: an anatomic study. J Hand Surg Am 17:767–772. https://doi.org/10.1016/0363-5023(92)90330-r
52	Parke (2004)	Parke WW (2004) Arteriovenous glomeruli of the human spinal cord and their possible functional implications. Clin Anat 17:558–563. https://doi.org/10.1002/ca.20046
53	Parry et al. (1988)	Parry SW, Ward JW, Mathes SJ (1988) Vascular Anatomy of the Upper Extremity Muscles: Plastic and Reconstructive Surgery 81:358–363. https://doi.org/10.1097/00006534-198803000-00007
54	Pedrini et al. (2024)	Pedrini FA, Innocenti M, Hassan K, Levin LS (2024) Surgical Feasibility Study on Cadaver for Vascularized Wrist Joint Transplantation. J Hand Surg Am 49:212–221. https://doi.org/10.1016/j.jhsa.2023.11.008

No.	Study	Full reference
55	Pérez-Cruz et al. (2024)	Pérez-Cruz JC, Macías-Duvignau MA, Reyes-Soto G, et al (2024) Latex vascular injection as method for enhanced neurosurgical training and skills. Front Surg 11:1366190. https://doi.org/10.3389/fsurg.2024.1366190
56	Plaisant et al. (1996)	Plaisant O, Sarrazin JL, Cosnard G, et al (1996) The lumbar anterior epidural cavity: the posterior longitudinal ligament, the anterior ligaments of the dura mater and the anterior internal vertebral venous plexus. Acta Anat (Basel) 155:274–281. https://doi.org/10.1159/000147816
57	Plaisant et al. (1998)	Plaisant O, Sarrazin JL, Gillot C, Lassau JP (1998) Technique for injection of the lumbar vertebral venous plexuses employed in anatomic, computed tomography and magnetic resonance imaging studies. Surg Radiol Anat 20:113–118. https://doi.org/10.1007/BF01628914
58	Poblete et al. (2021)	Poblete T, Casanova D, Soto M, et al (2021) Microsurgical Anatomy of the Anterior Circulation of the Brain Adjusted to the Neurosurgeon's Daily Practice. Brain Sci 11:519. https://doi.org/10.3390/brainsci11040519
59	Prat-Pradal et al. (2009)	Prat-Pradal D, Metge L, Gagnard-Landra C, et al (2009) Anatomical basis of transgluteal pudendal nerve block. Surg Radiol Anat 31:289–293. https://doi.org/10.1007/s00276-008-0445-z
60	Rees and Taylor (1986)	Rees MJW, Taylor GI (1986) A Simplified Lead Oxide Cadaver Injection Technique: Plastic and Reconstructive Surgery 77:141. https://doi.org/10.1097/00006534-198601000-00023
61	Renard et al. (2018)	Renard Y, Hossu G, Chen B, et al (2018) A guide for effective anatomical vascularization studies: useful ex vivo methods for both CT and MRI imaging before dissection. Journal of Anatomy 232:15–25. https://doi.org/10.1111/joa.12718
62	Rogers and Freeland (1976)	Rogers JH, Freeland AP (1976) Arterial vasculature of cervical skin flaps. Clin Otolaryngol Allied Sci 1:325–331. https://doi.org/10.1111/j.1365-2273.1976.tb00653.x
63	Safavi-Abbasi et al. (2016)	Safavi-Abbasi S, Komune N, Archer JB, et al (2016) Surgical anatomy and utility of pedicled vascularized tissue flaps for multilayered repair of skull base defects. J Neurosurg 125:419–430. https://doi.org/10.3171/2015.5.JNS15529
64	Sanan et al. (1999)	Sanan A, Aziz KMA, Janjua RM, et al (1999) Colored Silicone Injection for Use in Neurosurgical Dissections: Anatomic Technical Note. Neurosurgery 45:1267–1274. https://doi.org/10.1097/00006123-199911000-00058
65	Sen et al. (2011)	Sen T, Esmer AF, Acar HI, et al (2011) Arterial vascularisation of the anterior perforated substance. Singapore Med J 52:410–414
66	Serrato-Avila et al. (2022)	Serrato-Avila JL, Archila JAP, da Costa MDS, et al (2022) Microsurgical Anatomy of the Cerebellar Interpeduncular Entry Zones. World Neurosurg 166:e933–e948. https://doi.org/10.1016/j.wneu.2022.07.142
67	Shahbazi et al. (2021)	Shahbazi A, Feigl G, Sculean A, et al (2021) Vascular survey of the maxillary vestibule and gingiva-clinical impact on incision and flap design in periodontal and implant surgeries. Clin Oral Investig 25:539–546. https://doi.org/10.1007/s00784-020-03419-w
68	Shahbazi et al. (2020)	Shahbazi A, Pilsl U, Molnár B, Feigl G (2020) Detection of Vascular Pathways of Oral Mucosa Influencing Soft- and Hard Tissue Surgeries by Latex Milk Injection. Journal of Visualized Experiments (JoVE) e60877. https://doi.org/10.3791/60877
69	Shkarubo et al. (2018)	Shkarubo MA, Shkarubo AN, Dobrovolsky GF, et al (2018) Making Anatomic Preparations of the Human Brain Using Colored Silicone for Vascular Perfusion Staining (Technical Description). World Neurosurgery 112:110–116. https://doi.org/10.1016/j.wneu.2018.01.102
70	Singh et al. (2024)	Singh S, Chopra P, Necker F, et al (2024) Internal vascular anatomy of the human lacrimal gland: A protocol based on cadaver dissection and three-dimensional micro-computed tomography. Annals of Anatomy - Anatomischer Anzeiger 252:152207. https://doi.org/10.1016/j.aanat.2023.152207
71	Siriwittayakorn et al. (2024)	Siriwittayakorn W, Buranaphatthana T, Settakorn J, et al (2024) Simple modified silicone rubber injection technique in fresh cadaveric pelvis and extremities. Clinical Anatomy ca.24197. https://doi.org/10.1002/ca.24197
72	Smith et al. (2023)	Smith K, Ventre GJ, Palmisciano P, et al (2023) Brain Vasculature Color-Labeling Using the Triple-Injection Method in Cadaveric Heads: A Technical Note for Improved Teaching and Research in Neurovascular Anatomy. Oper Neurosurg (Hagerstown) 24:291–300. https://doi.org/10.1227/ons.0000000000000495

No.	Study	Full reference
73	Tanriover and Rhoton (2005)	Tanriover N, Rhoton AL (2005) The anteroinferior cerebellar artery embedded in the subarcuate fossa: a rare anomaly and its clinical significance. Neurosurgery 57:314–319; discussion 314-319. https://doi.org/10.1227/01.neu.0000166677.70797.5e
74	Urgun et al. (2014)	Urgun K, Toktas ZO, Akakin A, et al (2014) A novel, quick-prepared, colored silicone mixture for injecting into cerebral vasculature for neuroanatomical dissections: an anatomical technical note. Turkish Neurosurgery. https://doi.org/10.5137/1019-5149.JTN.12293-14.1
75	Vico et al. (1994)	Vico P, Coessens B, Heymans O, Vandeweyer E (1994) New mixture for simultaneous anatomical and radiological cadaver studies. Eur J Plast Surg 17:17–19. https://doi.org/10.1007/BF001764966
76	Wang et al. (2019)	Wang W-H, Lieber S, Mathias RN, et al (2019) The foramen lacerum: surgical anatomy and relevance for endoscopic endonasal approaches. J Neurosurg 131:1571–1582. https://doi.org/10.3171/2018.6.JNS181117
77	Xu et al. (2021)	Xu Y, Mohyeldin A, Doniz-Gonzalez A, et al (2021) Microsurgical anatomy of the lateral posterior choroidal artery: implications for intraventricular surgery involving the choroid plexus. J Neurosurg 135:1534–1549. https://doi.org/10.3171/2020.8.JNS202230
78	Zhao et al. (2002)	Zhao J-C, Chen C, Rosenblatt SS, et al (2002) Imaging the Cerebrovascular Tree in the Cadaveric Head for Planning Surgical Strategy. Neurosurgery 51:1222–1228. https://doi.org/10.1097/00006123-200211000-00018
